# Evaluating the measurement properties of patient-reported outcome measures for young adults with life-limiting conditions: A systematic review

**DOI:** 10.1177/02692163251340175

**Published:** 2025-06-19

**Authors:** Rachel L Chambers, Mevhibe B Hocaoglu, Irene J Higginson, Katherine E Sleeman, Lorna K Fraser

**Affiliations:** 1 Cicely Saunders Institute of Palliative Care, Policy & Rehabilitation, King’s College London, London, UK; 2 King’s College Hospital NHS Foundation Trust, London, UK; 3 Department of Women and Children’s Health, School of Life Course and Population Health, Faculty of Life Sciences and Medicine, King’s College London, London, UK

**Keywords:** Young adult, palliative care, patient reported outcome measures, outcome assessment, psychometrics

## Abstract

**Background:**

The number of young adults living with life-limiting conditions is increasing. This population requires palliative care responsive to their needs and preferences.

**Aim:**

To identify patient reported outcome measures developed, adapted and validated to assess the health outcomes of young adults (aged 18–25 years) living with life-limiting conditions. To examine their measurement properties and identify the most comprehensive, valid and reliable measures.

**Design:**

A systematic review and evaluation of measurement properties. PROSPERO ID (CRD42023443273).

**Data sources:**

MEDLINE; EMBASE; CINAHL; PsycInfo; AMED and Cochrane Library from inception to 03/07/2023. Searches were emented by hand-searching references.

**Results:**

Four thousand nine hundred twenty-two papers were identified. Five hundred and fifty-six full texts were assessed for eligibility. Thirty-five papers reporting 68 patient reported outcome measures were included. Most studies recruited young adults living with cancer (*n* = 29/35), we did not identify any studies with young adults living with complex neurodisability. Most measures (*n* = 61/68) were existing paediatric or adult patient reported outcome measures. Seven (*n* = 7/68) were young adult measures. Most were two-dimensional. The most assessed measurement properties were internal consistency, construct validity and structural validity. None of the measures were recommended for use as they did not meet sufficient criteria for content validity and internal consistency.

**Conclusion:**

There is a lack of multi-dimensional patient reported outcome measures for young adults living with life-limiting conditions, especially for non-cancerous conditions. Future studies may identify existing holistic measures developed for children or adults and adapt them for use with young adults. Studies should ask young adults and professionals about the relevance, comprehensiveness and comprehensibility of items.

**What is already known about the topic?**

- Patient reported outcome measures can facilitate communication, allowing patients to report their needs, symptoms and concerns to healthcare professionals and providing an opportunity to address unmet needs, leading to improved care.
- Young adults (aged 18–25 years) living with life-limiting conditions are a heterogeneous population, with a range of unique needs which are often unmet.

**What this paper adds?**

- There is a lack of multi-dimensional patient reported outcome measures for young adults living with life-limiting conditions, especially those with health conditions other than cancer.
- The measures identified have limited evidence of their psychometric properties.

**Implications for practice, theory or policy**

- Holistic patient reported outcome measures developed and validated for young adults with life-limiting conditions are needed.
- Future studies may identify existing holistic measures developed for children or adults and adapt them for use in young adults. Collaborating with young adults, their family members, clinicians and researchers during the development of patient-reported outcome measures (PROMs) is essential to ensure the items specifically address the unique physical, psychological, social and spiritual needs of young people.
- Clinicians play a vital role in ensuring patient reported outcome measures are relevant to real-world clinical settings, where they are used to support the care of young people by guiding treatment decisions and assessing well-being.
- PROMs should undergo rigorous evaluation of their measurement properties following the COSMIN (COnsensus-based Standards for the selection of health Measurement INstruments) guidelines, which ensure the tools are scientifically robust, reliable and valid for use with young people.
- Clear and consistent reporting of PROM development and validation studies, including transparent acknowledgment of limitations, is crucial. This allows clinicians and researchers to critically assess the quality of studies and select the most suitable and psychometrically sound measures tailored to young people’s needs.

## Background

Young adults (aged 18–25 years) living with life-limiting conditions are a heterogenous population.^1^ The population includes patients with childhood onset conditions who are transitioning into adult health services and may have cognitive and developmental delays^2^ and young adults diagnosed with a life-limiting condition in adulthood who require care from adult services.^1^

Internationally, the number of young adults living with life-limiting conditions or complex care needs is increasing,^3^ but there is a lack of global epidemiological data.^4^ This is further complicated by the inconsistent definition of the young adult age range between countries.^3^ In England, the number of people aged 18–25 years diagnosed with a life-limiting condition increased from 18,522 in 2009/2010 to 25,766 in 2017/2018, an increase of nearly 40%.^5^ Future projections estimate that by 2030 the prevalence of young people aged 14–25 years living with a life-limiting condition in England will be between 46.0 and 62.2 per 10,000, due to advances in medical technologies and improvements in the management of acute events.^5^ In the UK, congenital, neurological and respiratory conditions are more prevalent in young adults aged 18–25 years than oncology diagnoses.^5^ In Taiwan, the prevalence of life-limiting conditions in children and young adults (aged 0–25 years) increased from 45,311 in 2008 to 52,226 in 2017, with the highest prevalence in young adults aged 21–25 years, and oncology diagnoses being the most common.^6^ International trends in the incidence of cancer among adolescents and young adults (aged 15–39 years) showed an increase in cancer cases across 23 countries.^7^

The needs of young adults often differ to the needs of children and adults receiving palliative and end of life care.^8^ They are living with a life-limiting condition at a time of ‘physical, emotional, social and cognitive’ development^8^ meaning their needs are often broader than health, encompassing developmental needs including education, vocation, sexual relationships and living independently.^8,9^ Their illness may lead to ‘isolation and dependency’ on their families and healthcare services.^10^ Young adults require age-specific palliative care that is responsive to their needs and preferences at the end of life.^11^ Identifying unmet need is particularly important for young adults (aged 18–25 years) as they represent a ‘high-risk population who are underserved, with unique needs which are often unmet’.^12^

Patient reported outcome measures are standardised questionnaires to assess patients’ symptoms, quality of life and wellbeing.^13^ Patient reported outcome measures can facilitate communication between healthcare professionals, patients and family members.^2,3^ They allow patients to report their symptoms to health and social care professionals and provide an opportunity to address unmet needs, leading to improved care.^14^ Professionals should consider the psychometric properties of measures^15^ as well as factors such as diagnoses, geographical location, age, language, educational level and socioeconomic and cultural background of the target population when selecting outcome measures for use in research and clinical practice.^16^

To the best of our knowledge, there is no systematic assessment of the psychometric evidence of patient reported outcome measures which assess the health outcomes of young adults living with life-limiting conditions. This systematic review aims to identify patient reported outcome measures developed, adapted and validated to assess the health outcomes of young adults (aged 18–25 years) living with life-limiting conditions, to examine their measurement properties and to identify the most comprehensive, valid and reliable measures.

## Methods

This review was undertaken with a registered protocol (PROSPERO ID: CRD42023443273). This paper reports on review questions two and three in the registered protocol.

The review was carried out according to the COnsensus-based Standards for the selection of health Measurement INstruments (COSMIN) guidelines.^17^ It is reported in line with the Preferred Reporting Items for Systematic Reviews and Meta-Analysis (PRISMA) guidelines^18^ (Supplemental Material File 1: PRISMA Checklist).

## Searches

We searched MEDLINE; EMBASE; CINAHL (Cumulative Index to Nursing and Allied Health Literature); PsycInfo; AMED via Ovid and Cochrane Library from inception to 03/07/2023. Searches were supplemented by hand-searching reference lists of systematic reviews and included articles.

### Search strategy

The search was developed in MEDLINE using text words and subject headings with input and support from a librarian at King’s College London with expertise in systematic searches in medical research databases. The search strategy was adapted from Knighting et al’s (2021) systematic review of respite care and short breaks for young adults aged 18–40 years with complex healthcare needs.^19^ It follows the search strategy recommended by COnsensus-based Standards for the selection of health Measurement INstruments (COSMIN).^17^ No date limits were applied. The full search strategy can be accessed in Supplemental Material File 2: Search Strategy.

### Eligibility criteria

The eligibility criteria were based on those used in previous systematic reviews.^20,21^ Original, full-text peer-reviewed research studies, reporting the development, adaptation or validation of a patient reported outcome measure for young adults (aged 18–25 years) living with a life-limiting condition were included. Detailed inclusion and exclusion criteria are shown in Table 1.

**Table 1.** Inclusion/exclusion criteria.

Concept	Inclusion criteria	Exclusion criteria
Population	Study population that includes young adults (aged 18–25 years) living with a life-limiting condition (defined as individuals living with a condition for which there is no reasonable hope of cure which will cause them to die prematurely^22,23^). Studies of a mixed population will be considered for inclusion if: (1) the majority (⩾50%) of participants are young adults (aged 18–25 years) and (2) the relevant measures used for young adults are reported separately.	Study population solely comprising children or adults (i.e. aged <18 years or ⩾26 years). Percentage of young adults aged 18–25 years is not specified in the sample, or the mean or median fell outside of 18–25 years.
Concept	Patient reported outcome measures will be included. Papers reporting the development, adaptation or validation of patient reported outcome measures with young adults living with life-limiting conditions.	
Context	Patients in all settings in any country. Patient reported outcome measures developed for use in a research or clinical setting.	No study will be excluded based on the context.
Outcomes	Development, adaptation or validation of a patient reported outcome measure to assess the health outcomes of young adults living with life-limiting conditions. Evaluation of the measurement properties of patient reported outcome measures. Measurement properties include structural validity, internal consistency, cross-cultural validity, reliability, measurement error, criterion validity, hypothesis testing for construct validity and responsiveness.	
Study type	Original, full-text peer-reviewed research studies of all types. There are no publication date limits.	Systematic reviews, meta-analyses, case studies, descriptive, theoretical or clinical opinion articles, books, editorials and conference abstracts. Non-peer reviewed papers. Full-text not available.

The World Health Organisation defines adolescence as those aged 10–19 years and young people as those up to the age of 25 years.^24^ NHS England define young adults as those aged 18–25 years. This definition of young adults is based on biological evidence that the brain continues to develop until the mid-twenties,^25,26^ and people continue to develop socially and developmentally, as they transition from childhood to adulthood.^27^ This definition of young adult (those aged 18–25 years) was used for our eligibility criteria.

## Study selection

The search results were imported into Covidence (www.covidence.org). Duplicates were removed.

The first author (RLC) independently screened titles, abstracts and full texts against the inclusion criteria. A subset (20%) of papers were independently screened by a second reviewer (LKF). Conflicts were resolved by discussion.

## Data extraction

The first author (RLC) extracted data from each study into a standardised form (see Supplemental Material File 3: Data Extraction Form). Data extraction included: (1) study characteristics: author, year of publication, journal, geographic location, study design and characteristics of study sample (age, gender and disease); (2) characteristics of the patient reported outcome measure: patient reported outcome measure name, health outcome, mode of administration, recall period, length of instrument, number of items, response options, completion time, original language, available translations and validation methods used; (3) measurement properties including reliability, measurement error, criterion validity, structural validity, internal consistency, cross-cultural validity/measurement invariance, construct validity and responsiveness of the measure (see Table 2 for COnsensus-based Standards for the selection of health Measurement INstruments (COSMIN) definitions of measurement properties).^17^

**Table 2.** COnsensus-based standards for the selection of health measurement INstruments *(COSMIN) definitions of measurement properties.*^17^

Measurement property	Definition
Reliability	The degree to which the measurement is free from measurement error
Internal consistency	The degree of interrelatedness among the items
Test-retest	The extent to which scores for the patients (who have not changed) are the same for repeated measurements under several conditions over time
Measurement error	The systematic and random error of a patient’s score that is not attributed to true changes in the construct to be measured
Validity	The degree to which a patient reported outcome measure measures the construct(s) it purports to measure
Content validity	The degree to which the content of a patient reported outcome measure is an adequate reflection of the construct to be measured
Construct validity	The degree to which the scores of a measure are consistent with hypotheses (e.g. relationships to scores of other instruments, or differences between relevant groups) based on the assumption that the measure validly assesses the construct to be measured
Structural validity	The degree to which the scores of a measure are an adequate reflection of the dimensionality of the construct to be measured
Hypotheses testing	Item construct validity
Criterion validity	The degree to which the scores of a measure are an adequate reflection of a ‘gold standard’
Responsiveness	The ability of a measure to detect change over time in the construct to be measured

Information on interpretability of scores (distribution of scores, percentage of missing items, floor and ceiling effects and minimal important change/difference) were also extracted including information on feasibility of scores (patient/clinician comprehensibility, type and ease of administration, ease of standardisation, ease of score calculation, copyright, cost of instrument, required equipment, availability in different settings and requirements for approval). A second reviewer (MBH) independently checked the data extraction of all studies. Where it was unclear which measurement property papers were reporting on, the authors of this review made their own judgement based on the information provided. Conflicts were resolved by discussion.

### Data synthesis

The domains of the patient reported outcome measures identified were mapped onto a conceptual framework developed by Namisango et al.^28^ The framework consists of five categories: (1) physical, (2) psychological; (3) social; (4) spiritual and (5) other, based on the World Health Organisation’s definition of palliative care.^29^

We qualitatively summarised the measurement properties of the measures used to assess the health outcomes of young adults (18–25 years) living with life-limiting conditions.

### Assessment of methodological quality

We used the COnsensus-based Standards for the selection of health Measurement INstruments (COSMIN) Risk of Bias checklist^17,30,31^ to appraise the methodological quality of studies. The checklist comprises standards for patient reported outcome measure development and measurement properties (content validity, structural validity, internal consistency, cross-cultural validity/measurement invariance, reliability, measurement error, criterion validity, hypotheses testing for construct validity and responsiveness). Each were rated as ‘very good’, ‘adequate’, ‘doubtful’, ‘inadequate’ or ‘not applicable’. If authors did not specify which aspect of validity or reliability they were evaluating, the researchers made a judgement based on the methods used.^32^ The overall rating of each study was based on the ‘worst score counts’ principle. Two reviewers (RLC and MBH) independently assessed the methodological quality of included studies. Conflicts were resolved by discussion.

Next, the results of each study were rated against quality criteria developed by Terwee et al.^33^ The results of each measurement property, per study, were rated as sufficient (+), insufficient (−) or indeterminate (?).

### Recommending a patient reported outcome measure

After assessing the methodological quality of each of the studies we recommended patient reported outcome measures for use in accordance with COnsensus-based Standards for the selection of health Measurement INstruments (COSMIN) guidelines.^17^ Measures were classified into three categories: (1) Category A: patient reported outcome measures with evidence for sufficient content validity (any level) and at least low quality evidence for sufficient internal consistency; (2) Category B: Measures categorised not in A or C and (3) Category C: Measures with high quality evidence for an insufficient measurement property.^17^

## Results

**Figure 1.**
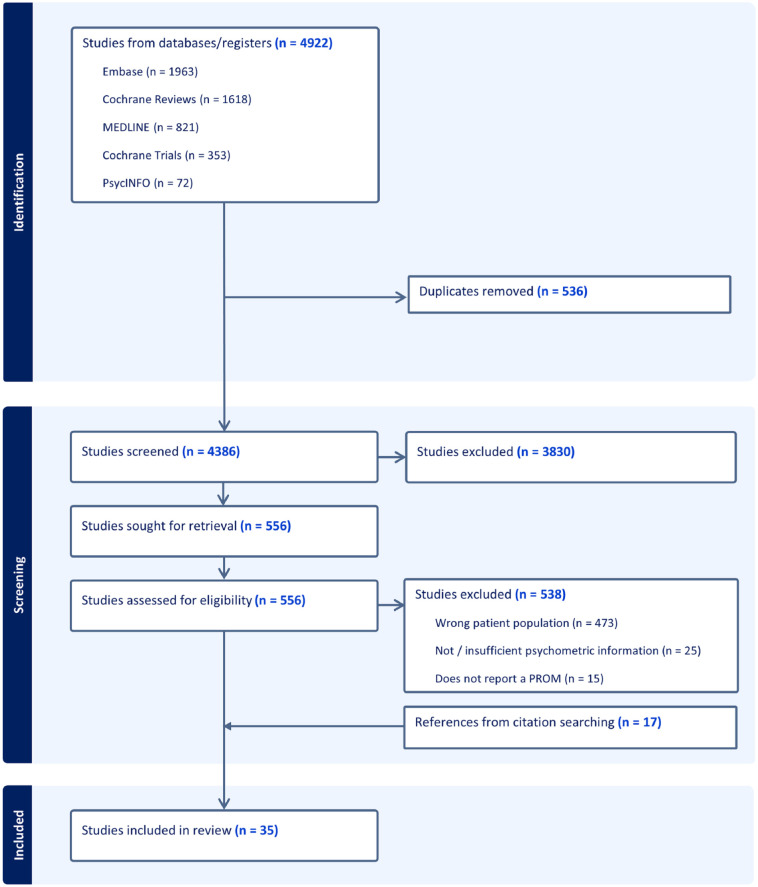
PRISMA flow chart.

After de-duplication, 4386 titles and abstracts were screened, 556 full-text papers were reviewed. Citation and reference searching identified an additional 17 papers for inclusion in the review. Thirty-five papers met the inclusion criteria (see Figure 1).

The main reason for exclusion was wrong patient population, specifically studies with outside of our age range of 18–25 years. Our search identified papers that developed, adapted or validated patient reported outcome measures for ages that spanned children, adolescents and young adults living with life-limiting conditions. In these studies, the age range was often 0–25 years or 14–30 years. This encompassed the age range for inclusion in our study (18–25 years), however, they were excluded from this review.

### Characteristics of included studies

Most studies included patients living with cancer (*n* = 29).^34–62^ One study included a mixed population, recruiting individuals living with cancer and blood disorders (*n* = 1).^63^ Other populations included young adults living with sickle cell disease (*n* = 2),^64,65^ cystic fibrosis (*n* = 1),^66^ spinal muscular atrophy (*n* = 1)^67^ and spina bifida (*n* = 1; see Table 3).^68^

**Table 3.** Characteristics of included studies.

Patient reported outcome measure(s)	Author	Country	Study Design	Aim(s)	Sample size	Age Mean (SD)	Gender	Disease	Race/ethnicity	Mode of PROM data collection
The Multidimensional Fatigue Symptom Inventory – Short Form (MFSI-SF) Brief Fatigue Inventory (BFI) PROMIS Fatigue Short Forms	Ameringer et al^64^	Not reported	Cross-sectional	(1) To describe fatigue; (2) To examine the relationship between fatigue and key biological and behavioural correlates, and personal and disease characteristics	60	22.5 (4.1)	Male 40%, Female 60%	Sickle Cell Disease	Not reported	Self-report
Hopkins Symptoms Checklist-25 (HSCL-25) Family APGAR SB Clinical Factors: Spina Bifida Severity and pain Child Attitude Towards Illness Scale	Bellin et al^68^	Not reported	Cross-sectional	To examine the relationship between multi-level ecological factors and psychological symptoms in young adults with spina bifida	61	21.05 (2.11)	Female 60.7%	Spina Bifida	Caucasian 77.0%	Self-report
Subjective Happiness Scale (SHS) Paediatric Health-Related Quality of Life (PedsQL) 4.0 Center for Epidemiologic Studies Depression (CES-D)	Bitsko et al^51^	Not reported	Cross-sectional	To evaluate two potential mediators to adolescent cancer survivors’ quality of life (QOL) and depressive symptomology	50	20.2	Male 50.0%; Female 48.0%	Cancer	Caucasian/White 82.0%; Minority 18.0%	Not reported
Fatigue Thermometer	Brand et al^47^	USA	Cohort study	To assess how well a single-item fatigue screen, consistent with recommendations by the National Comprehensive Cancer Network (NCCN), could be used as an accurate screening measure for clinically significant fatigue in childhood brain tumour survivors.	142	20.2 (4.8)	Male 47.0%; Female 53.0%)	Survivors of cancer	White/Caucasian 90.0%; African-American 3%; Hispanic 1.5%; Other or unknown 5.5%	Self-report
Health Competence Beliefs Inventory (HCBI) Brief Symptom Inventory-18 (BSI-18) Post-Traumatic Stress Disorder Checklist – Civilian Version (PCL-C)	Brier et al^39^	USA	Secondary analysis	(1) To use cluster analysis to identify profiles of subgroups of YA cancer survivors characterised by patterns of health competence beliefs; (2) To investigate demographic, psychosocial and health related correlates for each profile	119	21.6 (2.6)	Male 46.0%; Female 64.0%	Cancer	Caucasian 89.9%	Self-report and proxy reporting by professionals
Pain Thermometer	Chordas et al^48^	USA	Cohort and validation study	(1) To evaluate how well a single-dimension pain scale identifies clinically significant pain in childhood brain tumour survivors. (2) To determine if the pain thermometer could accurately identify survivors with clinically significant pain, with the aim of evaluating the pain thermometer for use in routine neuro-oncology follow-up care	99	Median 20	Male 46.5%; Female 53.5%	Survivors of cancer	White/Caucasian 86.8%; Asian/Pacific Islander 15.0%; African-American 2.0%; Hispanic 2.0%; Other 2.0%; Unknown 6.0%	Not reported
Cancer Needs Questionnaire – Young People (CNQ-YP)	Clinton-McHarg et al^46^	Australia	Validation study	(1) To establish face and content validity of the Cancer Needs Questionnaire – Young People (CNQ-YP); (2) To perform explanatory factor analysis, assess internal consistency and examine the test-retest reliability, discriminative validity, responsiveness and acceptability of the measure	139	Median 21	Male 36.0%; Female 64.0%	Cancer	Not reported	Self-report, postal
Health Competence Beliefs Inventory (HCBI) General Self-Efficacy Scale Perceived Health Competence Scale Brief Mood Rating Scale Post-Traumatic Stress Disorder Checklist – Civilian Version (PCL-C)	DeRosa et al^40^	Not reported	Validation study	(1) To develop a measure of health competence beliefs for adolescents and young adults; (2) To compare adolescent and young adult survivors and compare participants on these beliefs; (3) To provide preliminary evidence of validity by demonstrating the association of scores on this measure with other measures of beliefs, health and wellbeing	138 survivors; 130 comparison group	Survivors 20.3 (3.2)	Survivors female 56%	Cancer	Survivors: Caucasian 88.0%; African-American 4.0%; Asian 4%; Hispanic 2.0%; more than one race 1.0%	Self-report, in clinic or postal
PROMIS Self-Efficacy for Managing Symptoms Scale (PROMIS-S-E)	Erickson et al^36^	USA	Longitudinal, mixed methods	To examine the effects of a heuristic symptom assessment tool, the Computerised Symptom Capture Assessment Tool (C-SAT), on AYAs’ self-efficacy for symptom management, their self-regulation activities related to symptoms and negotiated collaboration, as operationalised by AYAs’ perceptions of communication with their providers about symptoms	85	20.9 (4.92)	Male 54%, Female 46%	Cancer	White 72%; African-American 12%; Native American/Alaska Native 2%; Asian 1%; Other or more than one race 13% Hispanic/Latino 21%; Non-Hispanic/Latino 79%	Self-report
Paediatric Quality of Life Inventory (PedsQL) 4.0 Generic Core Scale Adolescent Form Paediatric Quality of Life Inventory (PedsQL 3.0 Cancer Module Adolescent Form	Ewing et al^63^	Australia	Validation study	(1) To modify the existing adolescent forms of the PedsQL 4.0 Generic Core Scales and PedsQL 3.0 Cancer Module appropriate to AYA aged 16–25 years with cancer or a blood disorder; (2) To evaluate the reliability and validity of these modified forms	88 patients; 79 proxies	AYAs 16–19 years 54%; 20–25 years 46%	AYAs Female 58%	Cancer and blood disorders	Not reported	Self-report, in clinic or postal
Questionnaire on subjective wellbeing Strate Trait Anxiety Inventory – State subscale (STAI-S) Frankfurt Self-Concept Scale Questionnaire on Life Goals and Satisfaction with Life	Felder-Puig et al^43^	Not reported	Cross-sectional	To collect information about the psychosocial situation of young patients with multimorbidity therapy for bone cancer	60	23.5 (4.3)	Male 56.7%; Female 43.4%	Cancer	Not reported	Not reported
Health-Promoting Lifestyle Profile II (HPLP-II) The Ferrans and Powers Quality of Life Index (QLI): Cancer III Version	Finnegan et al^52^	USA	Cross-sectional	(1) To identify subgroups of ACC-survivors using self-reports of frequency, severity and distress ratings for eight symptoms (lack of energy, worry, pain, difficulty sleeping, feeling irritable, feeling nervous, difficulty concentrating and feeling nervous, difficulty concentrating and feeling sad) and factors that were likely to predict subgroup membership (chronic health conditions); (2) To determine the extent to which satisfaction with QoL varies across the subgroups	71	Median 23	Female 71.0%	Cancer	White 96.0%; Hispanic 9.0%	Self-report, online
Hospital Anxiety and Depression Scale (HADS) The Impact of Events Scale – Revised (IES-R) Metacognitions Questionnaire-30 (MCQ-30)	Fisher et al^42^	UK	Cross-sectional	To investigate whether metacognitive beliefs are associated with emotional distress and trauma symptoms in adolescents and young adults (AYA) survivors of cancer independent of known covariates, including current physical health difficulties	87	20.4 (2.0)	Male 47.0%; Female 53.0%	Cancer	Not reported	Self-report, in clinic or postal
The Post-Traumatic Diagnostic Scale (PDS) The Multidimensional Scale of Perceived Social Support (MSPSSI)	Ganz et al^53^	Israel	Descriptive, correlational	To investigate the prevalence, symptom, severity and risk factors associated with PTSD in survivors of childhood cancer	70	23.4 (4.3)	Male 54.0%; Female 46.0%	Cancer	Not reported	Self-report, in clinic
AYA Needs Assessment & Service Bridge (NA-SB)	Haines et al^44^	Not reported	Validation study	(1) To facilitate a systematic and patient-centred approach to delivering services to address the unmet needs of AYAs with cancer by developing the AYA Needs Assessment & Service Bridge (NA-SB). (2) To optimise the usability and usefulness of NA-SB using user-centred design.	Usability testing *n* = 99; Cognitive interviews n=5; Concept mapping *n* = 26; Design team prototype workshop *n* = not reported	Usability testing 24.2 (4.0)	Usability testing: Females 78.6%; Males 21.4%; Transgender 2.9%. Cognitive interviews: Male 20.0%; Female 80,0%	Cancer.	Usability testing: Non-Hispanic/White 70.0%; Hispanic (all races) 14.3%; Other 7.1%; Hispanic American Indian/Alaska Native 4.3%; Non-Hispanic Asian or Pacific Islander 2.9%; Non-Hispanic Black 1.4%	Self-report, online
Paediatric Quality of Life Inventory (PedsQL) Young Adult Version Hopkins Symptom Checklist-10 (HSCL-10)	Halvorsen et al^41^	Norway	Cross-sectional	To examine late effects in YA survivors of childhood cancer on self-reported HRQOL and distress and the association of these effects with treatment modalities, education and demographic factors	Survivors 91. Controls 223.	Survivors 24.7 (2.8)	Survivors. Females 61.5%	Cancer	Not reported	Not reported
PROMIS Pain Interference Short Forms PROMIS Fatigue Short Form	Hildenbrand et al^65^	USA	Validation study	To report preliminary psychometric properties (reliability and validity) of PROMIS (Pain Interference and Fatigue) assessments among emerging adults with sickle cell disease	45	20.6 (1.7)	Male 46.7%; Female 53.3%	Sickle Cell Disease	African-American 100%	Self-report, online
The Cancer Assessment for Young Adults – Testicular (CAYA-T)	Hoyt et al^59^	USA	Validation study	To establish a conceptual model and measurement instrument for assessment of HRQOL in young men with testicular cancer	171	25.2 (3.3)	Male 100%	Cancer	White (Non-Hispanic) 46.2%; Hispanic/Latino 38.0%; Asian 10.5%; Native American/Alaska Native 2.9%; African American/Black 1.2%; Other 1.2%	Self-report, in-person or online
JenAbdomen-CF Score	Jaudszus et al^66^	Germany	Validation study	(1) To examine whether the CFAbd-Score, a novel questionnaire consisting of 28 items, meets the requirements (validity and reliability) for a patient-reported outcome measure according to US Food and Drug Administration recommendations; (2) To confirm the conceptual framework and to assess the validity and reliability of the questionnaire	116 patients; 102 controls	Patients 23.3	Patients: Male 45.7%; Female 54.3%	Cystic Fibrosis	Not reported	Self-report, paper
Multidimensional Fatigue Inventory (MFI-20) Center for Epidemiologic Studies Depression Scale (CES-D)	Langeveld et al^61^	Netherlands	Cross-sectional	To assess the level of fatigue in young adult survivors of childhood cancer	Patients 416. Controls 1026	Patients 24.0 (5.2)	Patients: Male 52%; Female 48%	Cancer	Not reported	Self-report, in-clinic with investigator or postal
Spinal Muscular Atrophy – Health Index (SMA-HI)	Mazzella et al^67^	USA	Validation study	To provide information on the validity of the instrument in adolescent and young adults with SMA. To provide information regarding what symptomatic areas are most important to adolescents.	88	12–17 years 44%; 18–25 years 56%	Male 64.0%; Female 36.0%	Spinal Muscular Atrophy	White 79.0%; Black/African American 8.0%; Hispanic/Latino 5.0%; Asian Pacific Islander 2.0%; Other 6.0%	Self-report, online
Posttraumatic Stress Disorder Checklist (PCL-S) The Kessler Psychological Distress Scale (K10) The Multidimensional Scale of Perceived Social Support (MSPSSI) AYA Life Impact Checklist	McCarthy et al^62^	Australia	Cross-sectional	(1) To describe the prevalence of psychological distress (anxiety and depression) with PTSS in Australian AYAs with cancer and their parents, within 2 years following diagnosis; (2) To examine demographic illness/treatment and psychosocial variables associated with psychological distress	Patients 196. Parents 204	Patients 21.6 (3.1)	Patients. Male 51.0%; Female 49.0%	Cancer	Not reported	Self-report
Young Adult Psychosocial Assessment Strategy (YA-PAS)	McGrady et al^50^	USA	Validation study	(1) Phase 1. To conduct cognitive interviews with young adults with cancer to assess YA-PAS complexity, reliability and applicability and inform measure refinement; (2) Phase 2. To conduct a preliminary evaluation of YA-PAS reliability, validity, feasibility and acceptability among young adults who completed active cancer treatment, a group selected for the initial phase of testing as they comprise the vast majority of young adults with a history of a cancer diagnosis	Phase 1 *n* = 20. Phase 2: 100	Phase 1 22.9 (4.4). Phase 2 26.4 (5.7)	Phase 1. Male 60.0%; Female 40.0%. Phase 2 Male 39.0%; Female 58.0%; Other 1.0%	Cancer	Phase 1. White and non-Hispanic 85.0%; African-American or Black and Non-Hispanic 5.0%; White Hispanic 10%. Phase 2. White 86%; Black or African American 7.0%; Multiracial 2.0%; Asian 1.0%; Hawaiian or Pacific Islander 1.0%	Self-report, online
Symptom Checklist-90 Revised (SCL-90) Adult Behaviour Checklist (ABCL) Behaviour Rating Inventory of Executive Function – Adult Version (BRIEF-A) Fatigue Questionnaire (FQ)	Puhr et al^55^	Norway	Cross-sectional	(1) To explore the presence and degree of self-reported long-term executive dysfunction, psychological and emotional problems and fatigue in physically well-functioning PBT survivors as they enter into adulthood compared to a group of healthy controls matched for age, sex and education; (2) To investigate more specifically the presence of executive dysfunction in PBT survivors compared to healthy controls by examining whether this population has a specific profile of self-reported EF with respect to the hot and cool aspects of EF; 3. To explore the consistency between self and informant reports in this adult PBT survivor sample in order to address the possible problems of lowered awareness of deficits; 4. To explore how levels of self-reported executive dysfunction, psychological and emotional problems and fatigue are associated with tumor-related fatigue, treatment-related factors and medical late effects	Patients 114. Controls 169.	Patients 23.4 (3.5)	Patients. Female 57.9%	Survivors of cancer.	Not reported	Self or proxy report, postal
Cancer Distress Scales for Adolescents and Young Adults (CDS-AYA)	Rae et al^58^	Canada	Validation study	To determine cut points for clinical application of the CDS-AYA to screen for distress in the AYA cancer population	453	15–19 years *n* = 116; 20–29 years *n* = 205; 30–39 years *n* = 132	Male 55.4%; Female 44.4%; Not reported 0.2%	Cancer	Not reported	Self-report, paper or online
Cancer Distress Scales for Adolescents and Young Adults (CDS-AYA) Edmonton Symptom Assessment Scale-Revised (ESAS-r) Distress Thermometer Hospital Anxiety and Depression Scale (HADS)	Rae et al^56^	Canada	Validation study	To compare the Cancer Distress Scale for Adolescents and Young Adults (CDS-AYA) – Emotional and Impact Scales – with the Edmonton Symptom Assessment Scale-revised (ESAS-r), Hospital Anxiety and Depression Scale (HADS) and the National Comprehensive Cancer Network Distress Thermometer (NCCN DT) for use as a patient-reported outcome – performance measure (PRO-PM) for AYA cancer care	Field test *n* = 515; Test-retest *n* = 86; Included in the analysis *n* = 421	Analysis sample. 15–19 years 24.5%; 20–29 years 46.1%; 30–39 years 29.5%	Analysis sample. Male 55.6%; Female 44.4%	Cancer	Caucasian 59.4%; Asian 11.3%; Chinese 8.5%; South Other 20.4%; Not reported 0.4%	Self-report
Mishel Uncertainty in Illness Scale – Community (MUIS-C) State Trait Anxiety Inventory – State subscale (STAI-S) Posttraumatic Stress Disorder Reaction Index Posttraumatic Growth Inventory (PTGI) Growth Through Uncertainty Scale (GTUS) Health Promoting Lifestyle II	Santacroce et al^35^	Not reported	Pilot Randomised Controlled Trial	(1) To assess the feasibility of a randomised controlled trial of a telephone-delivered coping skills training (CST) intervention in terms of recruitment, retention and timeline, as well as the performance of the study measures; (2) to demonstrate trends in change on outcomes within the context of a small pilot study	20 AYAs	21.0 (3.7)	Female 52%	Cancer	White 85%, Hispanic 5%, Asian 5%, >1 race 5%	Self or proxy report, telephone
Functional Assessment of Cancer Therapy–General scale (FACT-G)	Skaczkowski et al^34^	Australia	Cross-sectional	To examine the relationship between the cancer care experiences of adolescents and young adults and their quality of life	209	21 (3.04)	Female 57.5%	Cancer	Not reported	Self-report, paper or online
PROMIS v2.0 Brief Profile Sex FS	Sopfe et al^60^	USA	Qualitative, validation study	To evaluate novel use of the PROMIS SexFS Brief in Adolescent CCS (<18 years) as well as to confirm acceptability, response process and content validity and usefulness specifically in emerging adult CCS (18–24 years)	24	Median 21	Male 41.7%; Female 58.3%	Cancer	White 79.2%; Asian 8.3%; Black/African American 8.3%; Other 4.2%	Self-report, online
Perceived Barriers Scale	Strauser et al^49^	USA	Validation study	To construct an instrument, the Perceived Barriers Scale, that has both clinical and research application related to career and employment issues of paediatric brain tumour survivors	110	23.1 (3.4)	Male 47.3%; Female 52.7%	Survivors of cancer	Caucasian 90.0%; Hispanic 3.6%; Asian or other Pacific Islander 3.6%; African-American 2.7%	Self-report, in clinic or postal
Posttraumatic Stress Disorder Checklist – Civilian Version (PCL-C) Chronic Disease Self-Efficacy Scale (Adapted)	Taylor et al^37^	UK	Cross-sectional	(1) To determine the incidence of PTSD in a UK population of young adult survivors of childhood cancer; (2) to determine relative contributions of demographic, medical and self-reported variables to PTSD and (3) to determine associations between PTSD and self-efficacy	108	20.8 (3.8)	Female 52.8%	Cancer	Not reported	Self-report, paper or online
Cancer Distress Scales for AYA (CDS-AYA)	Tsangaris et al^57^	Cananda	Validation study	To adapt the Australian AYA oncology and survivorship distress screening tools for use in Canada	Cognitive interview: *n* = 45; Field-testing *n* = 515; Test-retest *n* = 86; 4-month follow-up *n* = 67	Cognitive interview 15–19 years 42.2%; 20–29 years 42.2%; 30–39 years 15.6%. Field-testing 15–19 years 24.7%; 20–29 years 43.9%; 30–39 years 31.4%	Cognitive interview Male 51.1%; Female 48.9%. Field-testing Male 56.1%; Female 43.7%; Missing 0.2%	Cancer	Field-testing. Caucasian 59.4%; Asian 11.3%; Chinese 8.5%; South Other 20.4%; Not reported 0.4%	Self-report, paper or online
PROMIS Satisfaction with Social Roles and Activities – Short Form 8a (v2.0) PROMIS Ability to Participate in Social Roles and Activities – Short Form 8a (v2.0) PROMIS Social Isolation – Short Form 8a (v2.0) PROMIS Self-Efficacy for Managing Social Interactions – Short Form 8a (v1.0)	Walsh et al^38^	USA	Cross-sectional	(1) To examine if young adult cancer patients in the process of completing active treatment experienced social isolation, impairment in their ability to participate in social roles and activities, satisfaction with social roles and responsibilities and/or reduced self-efficacy for managing social interactions; (2) To assess if there were significant changes on measures of social isolation, ability to participate in social roles and activities, self-efficacy for managing social interactions and satisfaction with social roles and activities between the completion of active treatment and 3 months later	13	22.5 (2.5)	Male 53.9%; Female 46.2%	Cancer	Not reported	Self-report, postal
Quality of Life – Cancer Survivors (QOL-CS) 3 Worry Measures: (1) Cancer-specific worries; (2) Worries about general psychosocial issues; (3) General health worries	Zebrack and Chesler^45^	USA	Validation study	(1) To explore the underlying factor structure of an existing psychometric instrument designed to evaluate global and dimensional aspects of quality of life; (2) To evaluate the instrument’s reliability and validity, and thus its utility, in a sample of young adult survivors of childhood cancer, many of whom have entered their second decade of survivorship	177	21.8 (3.3)	Male 42.6%; Female 57.4%	Cancer	Caucasian 94.0%	Self-report, in-person or postal
Pittsburgh Sleep Quality Index Short Form-12 Brief Symptom Inventory-18 (BFI-18)	Zhou et al^54^	Not reported	Cohort study	(1) To describe the prevalence of clinically significant insomnia symptoms in this population; (2) to identify relationships between medical, physical and psychosocial patient characteristics with the presence/absence of clinically significant insomnia symptoms and (3) To evaluate the frequency with which sleep issues were documented during a neuro-oncology survivorship medical visit	98	23.3	Males 53.0%; Female 45.0%	Survivors of cancer	Not reported	

The population included in studies were predominantly from White ethnic groups. Sixteen studies did not report on race or ethnicity when describing the demographics of their sample. There was limited information on socioeconomic status including income, education and occupational status.

The 35 included studies reported the use of 68 patient reported outcome measures. Sixty one of these were existing paediatric, adolescent or adult patient reported outcome measures used and validated with young adults living with life-limiting conditions. Of these 61 measures, six were adapted or modified for use with young adults living with life-limiting conditions.^35,44,56–58,62,63^ Of the 68 measures identified, one measure was an existing young adult patient reported outcome measure . Of the 68 measures, 6 were new young adult patient reported outcome measures developed specifically for this population.^40,46,49,50,59,66^

Of the 68 patient reported outcome measures identified, 57 were generic measures and 11 were disease specific. Of these disease-specific measures most were developed for cancer populations (*n* = 8),^34,45,46,52,56–59,63^ followed by cystic fibrosis (*n* = 1),^66^ spinal muscular atrophy (*n* = 1)^67^ and spina bifida (*n* = 1).^68^ Most measures were two-dimensional, some were unidimensional (see Table 4). A minority of patient reported outcome measures (*n* = 3)^45,52,59^ for young adults living with cancer were shown to assess all domains of palliative care (physical, psychological, social and spiritual domains) when mapped onto Namisango et al’s^28^ conceptual framework (see Table 4).

**Table 4.** Patient reported outcome measures, conditions and domains.^28^

Patient reported outcome measure	Condition(s)	Domains				
		Physical 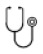	Psychological 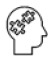	Social 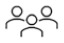	Spiritual 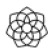	Other 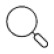
Three worry measures: (1) cancer-specific worries; (2) worries about general psychosocial issues and (3) general health worries^45^	Cancer		Worry	Worry		
Adult Behaviour Checklist (ABCL)^55^	Cancer		Emotional and behavioural problems			
AYA Life Impact Checklist^62^	Cancer		Self-image; identity; control over future	Work/education outlook; family relationships; interpersonal plans		
AYA Needs Assessment and Service Bridge^44^	Cancer	Physical health; sexual and reproductive health; health behaviours and wellness	Emotional health	Work; education; peer support and programming		Information; cancer centre team; finances and everyday needs
Behaviour Rating Inventory of Executive Function – Adult Version (BRIEF-A) ^55^	Cancer		Behaviour regulation; Metacognitive index; Global executive composite			
Brief Fatigue Inventory (BFI)^64^	Sickle Cell	Severity of fatigue		Interference of daily function		
Brief Mood Rating Scale^40^	Cancer		Positive and negative affect			
Brief Symptom Inventory – 18 (BSI-18)^29,54^	Cancer		Depression; Anxiety; Somatic symptoms			
Cancer Distress Scales for Adolescents and Young Adults (CDS-AYA)^56–58^	Cancer	Impact of cancer: physical	Impact of cancer: emotional, cognitive, cancer worry, mood			
Cancer Needs Questionnaire – Young People (CNQ-YP)^46^	Cancer		Feelings	Relationships; work, education; daily life		Cancer treatment centre; cancer treatment staff; information
Center for Epidemiologic Studies Depression Scale (CES-D)^51,61^	Cancer		Depressive symptomology			
Child Attitude Towards Illness Scale^68^	Spina Bifida		Feeling and attitudes about a health condition			
Chronic Disease Self-Efficacy Scale (Adapted)^37^	Cancer		Confidence in implementing tasks			
Distress Thermometer^56^	Cancer		Distress			
Edmonton Symptom Assessment Scale – Revised (ESAS-r)^56^	Cancer	Pain; tiredness; drowsiness; nausea; lack of appetite; shortness of breath	Depression; anxiety; wellbeing			Other
Family APGAR^68^	Spina Bifida			Family functioning (adaptation, partnership, growth, affection, roles)		
Fatigue Questionnaire (FQ)^55^	Cancer	Physical fatigue	Mental fatigue			
Fatigue Thermometer^47^	Cancer	Severity of fatigue				
Frankfurt Self-Concept Scale^43^	Cancer		General efficiency; coping with problems; confidence in own behaviours and decisions; self-esteem; vulnerability and mood	Steadfastness in groups; ability to mix with others; esteem by others; irritation caused by others; feeling towards and relations with others		
Functional Assessment of Cancer Therapy – General Scale (FACT-G)^34^	Cancer					Health-related quality of life
General Self-Efficacy Scale^40^	Cancer		Confidence in handling stressful situations			
Growth Through Uncertainty Scale (GTUS)^35^	Cancer		Continual uncertainty; acceptance of the situation; negative consequences		New view of life	
Health Competence Beliefs Inventory (HCBI)^40,40^	Cancer		Beliefs about health and wellbeing; health perceptions; Cognitive competence; autonomy			Satisfaction with healthcare
Health Promoting Lifestyle Profile II (HPLP-II)^35,52^	Cancer					Frequency of enacting specific health behaviours
Hopkins Symptom Checklist – 10 (HSCL-10)^41^	Cancer		Depression; Anxiety			
Hopkins Symptom Checklist – 25 (HSCL-25)^68^	Spina Bifida		Depression; Anxiety			
Hospital Anxiety and Depression Scale (HADS)^42,56^	Cancer		Anxiety; Depression			
Impact of Events Scale – Revised (IES-R)^42^	Cancer		Intrusions; avoidance; hyper-arousal			
JenAbdomen-CF Score^66^	Cystic Fibrosis	Pain symptoms; Reflux symptoms				Quality of life
Metacognitions Questionnaire – 30 (MCQ-30)^42^	Cancer		Metacognitive beliefs; Cognitive confidence; Need to control thoughts; Cognitive self-consciousness			
Mishel Uncertainty of Illness Scale – Community (MUIS-C)^35^	Cancer		Illness uncertainty			
Multidimensional Fatigue Inventory (MFI-20)^61^	Cancer	Physical fatigue	Mental fatigue; motivation	Reduced activities		
Paediatric health-related quality of life (PedsQL) 4.0^51^	Cancer					Health-related quality of life
Paediatric Quality of Life Inventory (PedsQL) 3.0 Cancer Module Adolescent Form^63^	Blood disorders Cancer	Pain and hurt; nausea	Procedural anxiety; treatment anxiety; worry; cognitive problems; perceived physical appearance			Communication
Paediatric Quality of Life Inventory (PedsQL) 4.0 Generic Core Scales Adolescent Form^63^	Blood disorders Cancer	Physical health	Emotional health	Social; school		
Paediatric Quality of Life Inventory (PedsQL) Young Adult version^41^	Cancer	Physical	Emotional	Social; work/school functioning		
Pain Thermometer^48^	Cancer	Pain intensity				
Perceived Barriers Scale^49^	Cancer			Career development and employment		
Perceived Health Competence Scale^40^	Cancer		Perceived self-efficacy to positive health outcomes			
Pittsburgh Sleep Quality Index^54^	Cancer	Sleep quality and disturbance				
Post Traumatic Growth Inventory (PTGI)^35^	Cancer		Personal growth	Relating to others	Spiritual changes; appreciation for life	
Post Traumatic Stress Disorder Reaction Index [36]	Cancer		Reexperiencing; hyperarousal; avoidance/numbing			
Post-Traumatic Stress Disorder Checklist – Civilian Version (PCL-C)^37,39,40^	Cancer		Symptoms of post-traumatic stress disorder			
Posttraumatic Stress Disorder Checklist (PCL-S)^62^	Cancer		Posttraumatic stress symptoms			
PROMIS Ability to Participate in Social Roles and Activities – Short Form 8a (v2.0)^38^	Cancer			Satisfaction with social roles and activities		
PROMIS Fatigue Short Form^64,65^	Sickle Cell	Impact and experience of fatigue				
PROMIS Pain Interference Short Form^65^	Sickle Cell			Interference with daily activities		
PROMIS Satisfaction with Social Roles and Activities – Short Form 8a (v2.0)^38^	Cancer			Satisfaction with social roles and activities		
PROMIS Self-Efficacy for Managing Social Interactions – Short Form 8a (v1.0)^38^	Cancer			Self-efficacy to manage social interactions		
PROMIS Self-Efficacy for Managing Symptoms Scale (PROMIS S-E)^36^	Cancer		Confidence in managing symptoms			
PROMIS Social Isolation – Short Form 8a (v2.0)^38^	Cancer			Social isolation		
PROMIS v2.0 Brief Profile Sex FS^60^	Cancer	Sexual function				
Quality of Life – Cancer Survivors (QOL-CS)^45^	Cancer	Physical wellbeing	Psychological wellbeing	Social wellbeing	Spiritual wellbeing	
Questionnaire on Life Goals and Satisfaction with Life^43^	Cancer					Life goals
Questionnaire on subjective wellbeing^43^	Cancer		Positive attitude towards life; Depressive mood; Joy of life			
SB Clinical Factors: Spina Bifida Severity and Pain^68^	Spina Bifida	Severity and pain				
Short Form-12^54^	Cancer					Health-related quality of life
Spinal Muscular Atrophy – Health Index (SMA-HI)^67^	Spinal Muscular Atrophy	Disease burden related to function; Fatigue; Hand and finger strength; Pain; Respiratory function; Swallowing function; Sleep; Gastrointestinal function; Mobility and ambulation	Emotional health	Social performance; Activity participation; Social satisfaction;		
State-Trait Anxiety Inventory – State subscale (STAI-S)^36,43^	Cancer		Anxiety			
Subjective Happiness Scale (SHS)^51^	Cancer		Global subjective happiness			
Symptom-Checklist 90 Revised (SCL-90)^55^	Cancer		Satisfaction; Obsessive-Compulsive; Depression; Anxiety; Hostility; Phobic Anxiety; Paranoid Ideation; Psychoticism			
The Cancer Assessment for Young Adults – Testicular (CAYA-T)^59^	Cancer	Physical; sexual confidence; sexual functioning	Body image; positive masculine self-image; positive adult self-image; cognitive-emotional regulation; disclosure ability	Relationship maintenance; social connectedness; recreational pursuit	Spiritual stability; finding meaning	Healthcare confidence; goal navigation; goal facility; financial maintenance;
The Ferrans and Powers Quality of Life Index (QLI): Cancer III Versions^52^	Cancer	Health and physical function	Psychological	Social and economic; family	Spiritual	Quality of life
The Kessler Psychological Distress Scale (K10)^62^	Cancer		Global distress; anxiety; depression			
The Multidimensional Fatigue Symptom Inventory–Short Form (MFSI SF)^64^	Sickle Cell	Physical fatigue	Emotional fatigue			
The Multidimensional Scale of Perceived Social Support (MSPSSI)^53,62^	Cancer			Instrumental and emotional social support		
The Post-Traumatic Diagnostic Scale (PDS)^53^	Cancer		Experience of a traumatic event; length of suffering	Interference with daily life		
Young Adult Psychosocial Assessment Strategy (YA-PAS)^50^	Cancer	Self efficacy for symptom and medication management	Anxiety; depression; cognitive function; post-traumatic stress	Family stressors; support; social isolation		

Most measures (*n* = 44) assessed psychological outcomes. Some measured multiple psychological domains including: mood (*n* = 17),^35,39–43,45,50,51,54–58,61–63,68^ cognition (*n* = 7),^39,40,41,50,55–59,63^ Posttraumatic Stress Disorder (*n* = 7),^35,37,29,40,42,50,53,62^ feelings towards their health condition (*n* = 6)^35,36,39,40,56–58,68^; body/self-image (*n* = 3)^29,62,63^; emotional fatigue (*n* = 3)^55,61,64^; distress (*n* = 2),^56,62^ behavioural problems (*n* = 2),^55^ identity (*n* = 1),^62^ control over the future (*n* = 1)^62^; motivation (*n* = 1),^61^ psychoticism (*n* = 1),^55^ paranoid ideation (*n* = 1).^55^

Some patient reported outcome measures (*n* = 23) measured physical symptoms, some measured multiple physical domains. Eleven measures (*n* = 11) assessed general physical health.^41,44,45,50,52,56–29,63,66,67^ Other measures assessed fatigue (*n* = 7),^47,55,61,64,65,67^ pain (*n* = 6)^48,56,63,66–68^ sexual/reproductive function (*n* = 3),^44,59,60^ sleep (*n* = 2),^54,67^ respiratory symptoms (*n* = 2),^56,67^ nausea (*n* = 2),^56,63^ tiredness (*n* = 1),^56^ drowsiness (*n* = 1),^56^ lack of appetite (*n* = 1),^56^ and mobility (*n* = 1).^67^

Some patient reported outcome measures measured social outcomes (*n* = 21). Six measures assessed general social outcomes (*n* = 6):^35,41,52,59,63,67^ interference with daily activities (*n* = 5) ^46,53,61,64,65^; social support (*n* = 3),^44,50,53,62^ satisfaction with social roles/activities (*n* = 3)^38,67^; education (*n* = 5),^41,44,46,62,63^ career/employment (*n* = 3),^41,46,49^ activity participation (*n* = 2),^59,67^ family functioning (*n* = 2),^62,68^ ability to manage social interactions (*n* = 3),^38,43,59^ social isolation (*n* = 1)^38^ and worries about psychosocial issues (*n* = 1).^45^

A minority of measures (*n* = 5) assessed the spiritual needs of young adults living with life-limiting conditions. Most of these measures asked about general spiritual needs (*n* = 3),^45,52,59^ others (*n* = 2) asked about the individual’s perception of life.^35^ All measures that assessed spiritual needs were validated for young adults living with cancer.^35,45,52,59^

### Measurement properties

Some measures had multiple research studies reporting on their measurement properties. They were the PROMIS Fatigue Short Form,^64,65^ The Multidimensional Scale of Perceived Social Support,^53,62^ Hopkins Symptom Checklist,^41,68^ Health Competence Beliefs Inventory,^39,40^ State-Trait Anxiety Inventory,^43,64^ Center for Epidemiologic Studies – Depression,^51,61^ Hospital Anxiety and Depression Scale,^42,56–58^ the Brief Symptom Inventory^439,54^ and the Cancer Distress Scales for Adolescents and Young Adults (CDS-AYA).^56–59^

It was not possible to quantitatively pool results as studies were heterogenous; studies evaluating the same tools collected data from respondents with different medical conditions or used different methodologies (e.g. randomised controlled trials vs. cross sectional designs). Therefore, the data for each study are reported separately,^69,70^ categorised by each patient reported outcome measure (see Supplemental Material File 4: Patient reported outcome measure characteristics).

### Methodological quality

The methodological quality of the included studies is shown in Table 5. The following measurement properties were assessed in the included papers: content validity (*n* = 8), structural validity (*n* = 15), internal consistency (*n* = 73), test-retest reliability (*n* = 9), hypothesis testing for construct validity (*n* = 24) and responsiveness (*n* = 4). One study reported information on measurement error.^66^ Measurement invariance was assessed by one study evaluating the Cancer Distress Scale for Adolescents and Young Adults (CDS-AYA).^57^ Criterion validity was not reported in any of the studies included in this review. Most studies were limited by their small sample size which reduced the strength of the reported evidence. None of the studies reported information on all measurement domains

**Table 5.** COnsensus-based Standards for the selection of health Measurement INstruments (COSMIN) risk of bias^30^ and quality criteria^33^ assessments for each measurement property, per study.

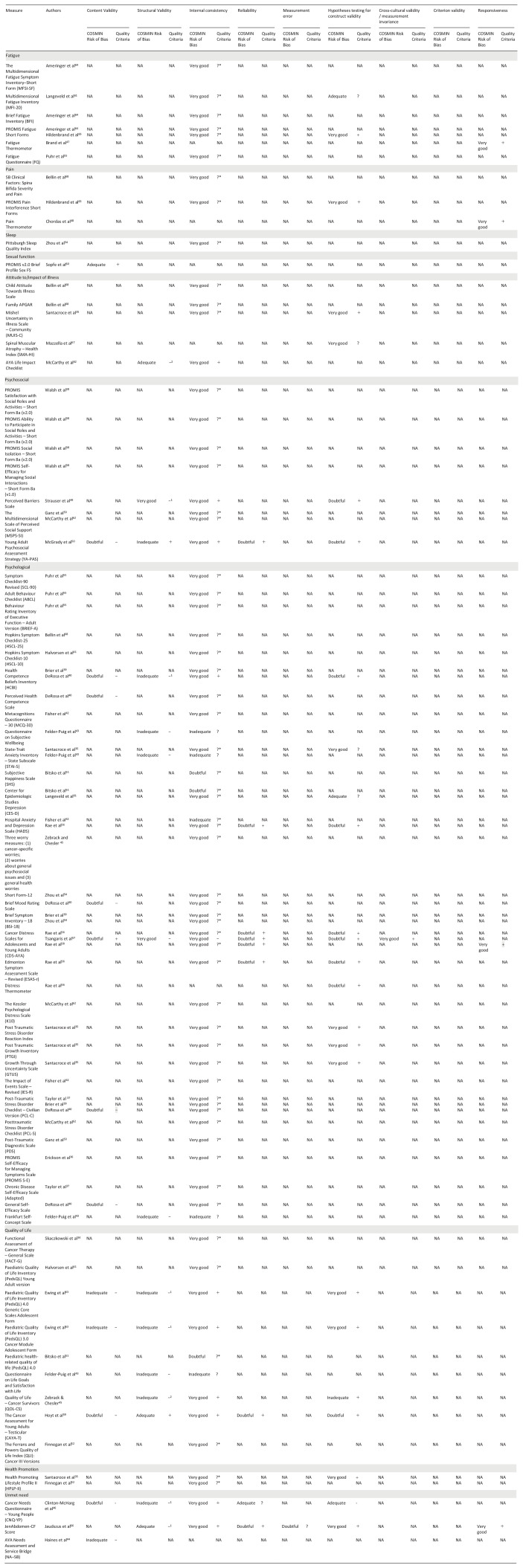

NA no information provided in the paper.

+ Sufficient.

? Indeterminate.

?* Rated indeterminate as there is no information on structural validity.

− Insufficient.

−1 insufficient. Exploratory factor analysis used. Quality criteria relates to confirmatory factor analysis.

−2 insufficient. Principal component analysis used. Quality criteria relates to confirmatory factor analysis.

The shaded rows group the measures by the outcomes assessed.

### Content validity

Content validity is the degree to which the content of a patient reported outcome measure is an adequate reflection of the construct to be measured.^30^ Eight studies reported the assessment of content validity. The methodological quality of most (*n* = 7) studies was considered ‘doubtful’. Of these, six studies consulted both healthcare professionals and patients on the relevance and understanding of the items included in the measure. In one study the methodological quality was considered ‘adequate’ for the components reported but they did not appear to consult healthcare professionals about the content of the patient reported outcome measure. Five studies assessed the measure for relevance, comprehensibility and comprehensiveness. Most provided little description of the qualitative methods used (e.g. whether skilled interviewers were used and their approach to data analysis).

### Structural validity

Structural validity is the degree to which the scores of a patient reported outcome measure are an adequate reflection of the dimensionality of the construct to be measured.^30^ Fifteen studies assessed this domain. Methodological quality was rated as ‘very good’ in two studies, ‘adequate’ in three studies and ‘inadequate’ in 10 studies. Studies of higher quality reported findings of confirmatory factor analysis. Most studies were graded as adequate/inadequate due to insufficient sample size as recommended by COnsensus-based Standards for the selection of health Measurement INstruments (COSMIN) guidelines.^18,30,31^

### Internal consistency

Internal consistency refers to the degree of interrelatedness among items.^30^ Most papers reported internal consistency. Thirty-two patient reported outcome measures were only assessed for internal consistency.^34,36–42,45,51–55,62,64,68^ The Fatigue Thermometer, Pain Thermometer, Distress Thermometer (single item measures) and the Spinal Muscular Atrophy – Health Index (SMA-HI) did not report the results of Cronbach’s Alpha. Most studies were assessed as having ‘very good’ methodological quality for internal consistency. Those that did not score ‘very good’ were downgraded as structural validity was not assessed or was deemed inadequate, in line with COnsensus-based Standards for the selection of health Measurement INstruments (COSMIN) guidance.^18,30,31,33^

### Test-retest reliability

Test-retest reliability is the extent to which scores for the patients (who have not changed) are the same for repeated measurements under several conditions over time.^30^ Nine studies reported on test-retest reliability. All were rated ‘doubtful’ as it was unclear whether patients were stable in the interim period, whether the time interval was appropriate, and whether test conditions were similar.

### Measurement error

Measurement error is the systematic and random error of a patient’s score that is not attributed to true changes in the construct to be measured.^30^ Only one study reported information on this domain.^66^ The methodological quality was rated as ‘doubtful’ as it was unclear whether patients were stable in the interim period, whether the time interval was appropriate, and whether test conditions were similar.

### Construct validity

Construct validity is the extent to which scores of an outcome measure are consistent with hypotheses.^30^ In most studies, the hypotheses were clearly defined or reported in the papers. Twenty-four studies reported results for construct validity. Methodological quality was mixed. Eleven studies were rated as ‘very good’, three were rated as ‘adequate’, nine were rated as ‘doubtful’ and one was rated as ‘inadequate’.

### Measurement invariance

Measurement invariance was assessed by one study. The methodological quality was rated as ‘very good’. All criteria were sufficiently described.^57^

### Responsiveness

Responsiveness is the ability of a patient reported outcome measure to detect change over time in a construct to be measured.^30^ Three studies compared the patient reported outcome measure to a gold standard, whilst one compared the patient reported outcome measure with other outcome measurement instruments. The methodological quality of all studies was ‘very good’.

## Recommended patient reported outcome measures

Most patient reported outcome measures included in this review were evaluated based on the results of a single validation study. Some measures had multiple studies reporting different measurement properties, but the populations studied were heterogeneous and did not allow us to quantitatively pool results. This limited our ability to make recommendations.

No study assessed all psychometric properties. Most patient reported outcome measures (*n* = 39/68) had evidence of one measurement property, the majority of which reported on internal consistency which was rated as ‘indeterminate’ as there was no evidence for structural validity. Sixteen patient reported outcome measures had evidence of two psychometric properties. The remaining measures (*n* = 13/68) reported three or more measurement properties.

All outcome measures included in this review were placed in Category B. They have potential to be recommended for use, but they require further research to assess their comprehensiveness, reliability and validity in the young adult population.^30^ None of the measures met the criteria to be placed into Category A as they do not meet sufficient criteria for content validity and internal consistency.

## Discussion

### Main findings/results of the study

Our review identified 68 patient reported outcome measures to assess the health outcomes of young adults (aged 18–25 years) living with life-limiting conditions. Of these measures, most were generic (not disease-specific). Most measures were validated in cancer populations, we did not identify any measures for young adults living with neurodisabilties. The majority were two-dimensional rather than holistic, focussing on the assessment of psychological symptoms such as mood and wellbeing. Most papers measured internal consistency. Few studies reported on content validity, measurement error, measurement invariance and responsiveness. No studies assessed criterion validity.

### What this study adds?

In young adults (aged 18–25 years) congenital, neurological and respiratory conditions are more prevalent than oncology diagnoses.^5^ Despite this, most measures we identified were psychometrically assessed in young adults living with cancer. There was a lack of measures developed, adapted or validated for young adults living with non-cancerous conditions. Specifically, there were no studies assessing the health outcomes of young adults living with complex neurodisability despite the large number of young adults affected by these conditions.

Most of the measures identified were generic, and not specific to a particular condition. Generic measures allow for comparisons of health outcomes across populations^71^ which may be beneficial for heterogenous groups such as young adults living with life-limiting conditions as the types of diagnoses and complexity varies.^1^ Alternatively, disease-specific measures allow for comparisons of health outcomes within a particular condition^71^ and can be sensitive to change when evaluating the effects of interventions or treatments^71,72^ but given the large number of different diseases within this population this makes it challenging to develop disease-specific measures. Therefore, this is not something that we think would be useful as the process of develop disease-specific measures would be complex and less efficient and the benefit to young adults living with life-limiting conditions would be limited.

A review by Contri et al^16^ explains that the validity of a patient reported outcome can be affected by the context in which they are applied. This includes ‘geographical location, age, language, educational level and socioeconomic and cultural background of the target population’.^16^ We found that most studies did not report educational level or socioeconomic characteristics of their sample. In line with a review of patient reported outcome measures for young adults living with cancer,^73^ we found that minority groups were underrepresented in most studies. Sixteen papers did not report on ethnicity or race when describing participant demographics. This makes it unclear whether the items or constructs measured by the patient reported outcome measure are meaningful, comprehensive or valid for different groups. The need for representation of racially and ethnically minoritised groups has been called for by Rhodes et al^74^ who recommend that descriptions of race and ethnicity are standardised across research, and researchers should ‘strive for diversity, equity and inclusion among research participants’. They suggest that quota sampling on race and ethnicity may facilitate this.^75^ This would help to ensure that outcome measures are comprehensive of the needs of diverse groups of young adults living with life-limiting conditions when implemented in clinical practice and research studies.

Most of the included papers did not comprehensively report the methods used to assess the reliability and validity of patient reported outcome measures. In some papers it was unclear which measurement property the authors were reporting on. In these circumstances, the authors of this review made their own judgement based on the information provided. This may explain the variation in methodological quality (COnsensus-based Standards for the selection of health Measurement INstruments (COSMIN) risk of bias assessment). This is consistent with previous research which identified that ‘studies examining measurement properties are often missing key information that allows the reader to determine the methods used, what the results are and what the research means for the evidence of the quality of a particular patient reported outcome measure’.^76^ This may also be explained by the publication dates of included studies which spanned the years of 1998 to 2021. During this period there have been numerous guidelines and recommendations for the reporting of validation studies.^76^ To standardise and facilitate the comprehensive reporting of validation studies, COnsensus-based Standards for the selection of health Measurement INstruments (COSMIN) guidelines should be utilised. This should improve the ability of reviewers to quality appraise measurement papers^76^ and will support the selection of patient reported outcome measures for use in clinical practice and research studies.

Internal consistency, construct validity and structural validity were the most assessed measurement properties. Three studies assessed responsiveness; it is therefore unclear whether the measures identified were responsive to change.^71^ This should be considered when implementing these tools in clinical practice or measuring the effects of an intervention. Content validity is the extent to which the content of a patient reported outcome measure is relevant, complete and comprehensive of the construct to be measured and for the target population and is considered to be the most important measurement property by COnsensus-based Standards for the selection of health Measurement INstruments (COSMIN).^17^ To meet COnsensus-based Standards for the selection of health Measurement INstruments (COSMIN) criteria to be recommended for use, a patient reported outcome measure requires at least sufficient evidence for content validity and internal consistency. We found insufficient evidence for content validity and internal consistency so all of the measures were placed in category B and have the potential to be recommended for use. Thirteen studies reported on content validity, but the quality of evidence for 12 of these studies was rated as insufficient. One patient reported outcome measure (PROMIS v2.0 Brief Profile Sex FS) had sufficient criteria for content validity but did not have any evidence for internal consistency.^60^ COnsensus-based Standards for the selection of health Measurement INstruments (COSMIN) guidance outlines that in circumstance where all patient reported outcome measures are placed in category B, the measure with best evidence for content validity can be provisionally recommended for use until additional evidence is provided.^17^ In this circumstance the patient reported outcome measure was very specific, only measuring sexual function, and was therefore not recommended for use.

For most of the measures identified it is unclear how young adults living with life-limiting conditions interpret the items, whether the items are perceived as relevant from their own experiences and whether the results are useful for clinical decision making.^71^ Future studies should take existing patient reported outcome measures, covering all domains of palliative care (physical, psychological, social and spiritual) and ask young adults (aged 18–25 years) and professionals (clinicians and researchers) about the relevance, comprehensiveness and comprehensibility of items in line with COnsensus-based Standards for the selection of health Measurement INstruments (COSMIN) guidance.^30^ Where young adults are unable to self-report, we recommend proxy-reporting by families or healthcare professionals where possible.

Most measures identified were two-dimensional either assessing physical and psychological outcomes, or psychological and social outcomes. Even within these dimensions, many measures only assessed one specific aspect, that is, physical fatigue. Only three patient reported outcome measures were holistic,^45,52,59^ assessing each domain of palliative care as defined by the World Health Organisation (physical, psychological, social and spiritual/existential)^29^ and notably these measures were all developed and validated in cancer populations. Future studies may identify existing holistic measures that have been developed for adults or children and adapt them for use in young adult populations.

### Strengths and limitations of the study

A strength of this review is that it adheres to the COnsensus-based Standards for the selection of health Measurement INstruments (COSMIN) methodology for systematic reviews of patient reported outcome measures. Our search strategy was broad and was not limited by language or date restrictions to capture all relevant papers. Our search identified papers that developed, adapted or validated patient reported outcome measures for ages that spanned children, adolescents and young adults living with life-limiting conditions. In these studies, the age range was often 0–25 years or 14–30 years. This encompassed the age range for inclusion in our study (18–25 years), however, the papers did not specify the percentage of young adults aged 18–25 years in their sample, or the mean or median age fell outside our inclusion criteria (18–25 years). They were therefore excluded from this review. There may be high quality studies that have adequately assessed the measurement properties of holistic patient reported outcome measures, but because of our strict inclusion criteria they were not included in this review, but they may still be relevant to the young adult population.

## Conclusion

We identified a number of one- and two-dimensional patient reported outcome measures for young adults living with life-limiting conditions. Most were validated in young adults living with cancer, with non-cancerous populations underrepresented, especially those with complex neurodisability. Future studies should examine existing patient reported outcome measures that cover all domains of palliative care (physical, psychological, social and spiritual) and assess their suitability for use in young adults. To standardise and facilitate the comprehensive reporting of validation studies, COnsensus-based Standards for the selection of health Measurement INstruments (COSMIN) guidelines should be utilised. This will support the selection of patient reported outcome measures for use in clinical practice and research studies.

## Supplemental Material

sj-docx-1-pmj-10.1177_02692163251340175 – Supplemental material for Evaluating the measurement properties of patient-reported outcome measures for young adults with life-limiting conditions: A systematic review Supplemental material, sj-docx-1-pmj-10.1177_02692163251340175 for Evaluating the measurement properties of patient-reported outcome measures for young adults with life-limiting conditions: A systematic review by Rachel L Chambers, Mevhibe B Hocaoglu, Irene J Higginson, Katherine E Sleeman and Lorna K Fraser in Palliative Medicine

sj-docx-2-pmj-10.1177_02692163251340175 – Supplemental material for Evaluating the measurement properties of patient-reported outcome measures for young adults with life-limiting conditions: A systematic review Supplemental material, sj-docx-2-pmj-10.1177_02692163251340175 for Evaluating the measurement properties of patient-reported outcome measures for young adults with life-limiting conditions: A systematic review by Rachel L Chambers, Mevhibe B Hocaoglu, Irene J Higginson, Katherine E Sleeman and Lorna K Fraser in Palliative Medicine

sj-docx-4-pmj-10.1177_02692163251340175 – Supplemental material for Evaluating the measurement properties of patient-reported outcome measures for young adults with life-limiting conditions: A systematic review Supplemental material, sj-docx-4-pmj-10.1177_02692163251340175 for Evaluating the measurement properties of patient-reported outcome measures for young adults with life-limiting conditions: A systematic review by Rachel L Chambers, Mevhibe B Hocaoglu, Irene J Higginson, Katherine E Sleeman and Lorna K Fraser in Palliative Medicine

sj-xlsx-3-pmj-10.1177_02692163251340175 – Supplemental material for Evaluating the measurement properties of patient-reported outcome measures for young adults with life-limiting conditions: A systematic review Supplemental material, sj-xlsx-3-pmj-10.1177_02692163251340175 for Evaluating the measurement properties of patient-reported outcome measures for young adults with life-limiting conditions: A systematic review by Rachel L Chambers, Mevhibe B Hocaoglu, Irene J Higginson, Katherine E Sleeman and Lorna K Fraser in Palliative Medicine
