# Supplementary material for: Evaluating the measurement properties of patient-reported outcome measures for young adults with life-limiting conditions: A systematic review
Source: Palliat Med. 2025 Jun 19;39(7):765–91. doi: 10.1177/02692163251340175 (PMC12227827; doi:10.1177/02692163251340175)
Supplement: sj-docx-4-pmj-10.1177_02692163251340175 – Supplemental material for Evaluating the measurement properties of patient-reported outcome measures for young adults with life-limiting conditions: A systematic review [file sj-docx-4-pmj-10.1177_02692163251340175.docx]

*Supplementary File 4: Patient reported outcome measure characteristics*

| **Measure** | **Studies included** | **Recall period** | **Number of items** | **Response options** | **Range of scores** |
| --- | --- | --- | --- | --- | --- |
| The Multidimensional  Fatigue Symptom Inventory–Short Form (MFSI-SF) | Ameringer et al. [55] | 1 month | 30 items. 5 subscales (general, physical, emotional, mental, vigor) | 5-point Likert scale. 0 (not at all) to 4 (extremely) | 0-20 |
| Multidimensional Fatigue Inventory (MFI-20) | Langeveld et al. [52] | Not reported | 20 items | 5-point scale | Not reported |
| Brief Fatigue Inventory (BFI) | Ameringer et al. [55] | 1 week | 10 items | 11-point numeric rating scale | Mean total score of the 9 items is calculated. |
| PROMIS Fatigue Short Forms | Ameringer et al. [55]  Hildenbrand et al. [56] | 1 week | 7 items | 5-point Likert scale ranging from “never” to “always” | A  total score was calculated by summing scores across  items. |
| Fatigue Thermometer | Brand et al. [38] | 1 week | 1 item | 10-point Likert scale and a visual analogue scale | 0-10 |
| Fatigue Questionnaire (FQ) | Puhr et al. [46] | 1 month | 11 items across 2 subscales | 0 to 3; duration of symptoms; dichotomised score | Not reported |
| SB Clinical Factors: Spina Bifida Severity and Pain | Bellin et al. [59] | 1 week | 5 items (4 for severity and 1 for pain) | Shunt status (1 = no, 2 = yes), myelomeningocele (1 = no, 2 = yes), lesion level (sacral = 1, lumbar = 2, thoracic = 3), ambulation status (no assistance = 1, needs assistive devices to walk = 2, wheelchair use = 3); Pain rated using a visual analogue scale 1= no pain to 10 = extreme amount of pain. | Severity total score 4-10. Pain total score 1-10. |
| PROMIS Pain Interference Short Forms | Hildenbrand et al. [56] | 1 week | 6 items | 5-point scale (1=not at all, 5=very much) | Not reported |
| Pain Thermometer | Chordas et al. [39] | 1 week | 1 item | 10-point Likert scale (0-3 mild pain, 4-6 moderate, 7-10 severe) and visual analogue scale | 0-10 |
| JenAbdomen-CF Score | Jaudszus et al. [57] | 2 weeks | 28 items across 5 domains | 6-point scale or 10-point scale with mood expressing faces | 0-100 |
| Pittsburgh Sleep Quality Index | Zhou et al. [45] | 1 month | 19 items | Not reported | Not reported |
| PROMIS v2.0 Brief Profile Sex FS | Sopfe et al. [51] | Not reported | 10 items for males and 14 items for females | Not reported | Not reported |
| Child Attitude Toward Illness Scale | Bellin et al. [59] | Not reported | 12 | 1-5 | 12-60. |
| Mishel Uncertainty in Illness Scale – Community (MUIS-C) | Santacroce et al. [26] | Not reported | 21 items | 5-point Likert scale | Not reported |
| AYA Life Impact Checklist | McCarthy et al. [53] | Not reported | 18 items | Checklist | Not reported |
| Family APGAR | Bellin et al. [59] | Not reported | 5 | 1-5 (1 = never, 5 = always) | 5-25 |
| PROMIS Satisfaction with social roles and activities – short form 8a (v2.0) | Walsh et al. [29] | Not reported | Not reported | Not reported | Not reported |
| PROMIS Ability to Participate in Social Roles and Activities—short form 8a (v2.0) | Walsh et al. [29] | Not reported | Not reported | Not reported | Not reported |
| PROMIS Social Isolation – Short form 8a (v2.0) | Walsh et al. [29] | Not reported | Not reported | Not reported | Not reported |
| PROMIS Self-Efficacy for Managing Social Interactions—short form 8a (v1.0) | Walsh et al. [29] | Not reported | Not reported | Not reported | Not reported |
| Perceived Barriers Scale | Strauser et al. [40] | Not reported | 12 items | 11-point Likert scale (0=not a barrier, 10=major barrier) | Not reported |
| The Multidimensional Scale of Perceived Social Support (MSPSSI) | Ganz et al. [44]  McCarthy et al. [53] | Not reported | 12 items | 1-7 | 12-84 |
| Young Adult Psychosocial Assessment Strategy (YA-PAS) | McGrady et al. [41] | Not reported. | 41 items across 9 domains | Not reported | Anxiety, depression, cognitive function, 4-20, Depression 4-20, cognitive functioning 4-20, post-traumatic stress 0-6, family stressors 0-7, support 0-10, social isolation 4-20, self-efficacy  for symptom management 4-20, and self-efficacy for medication management 4-20), with the substance use and life stressors items reconceptualized as non-scoring (in addition to the original non-scored areas of resources, educational/vocational status, and relationship status) |
| Symptom Checklist-90 Revised (SCL-90) | Puhr et al. [46] | 1 week | 90 items across 9 scales | 5 point Likert Scale | Not reported |
| Adult Behaviour Checklist (ABCL) | Puhr et al. [46] | Not reported | 164 items across 2 scales | 3-point scale | Three composite scores are generated: total problems, internalising problems (sum of the syndrome scales anxious/depressed, withdrawn, and somatic complaints) and externalising problems (sum of the syndrome scales aggressive behaviour, rule-breaking behaviour, and intrusive behaviour) |
| Behaviour Rating Inventory of Executive Function – Adult Version (BRIEF-A) | Puhr et al. [46] | 6 weeks | 75 items | 3-point scale | Not reported |
| Hopkins Symptom Checklist-25 (HSCL-25) | Bellin et al. [59] | Not reported | 25 items. 2 subscales (depression = 10 items, anxiety = 15 items) | Likert Scale. 1 = not at all to 4 = extremely | Depression 15-60. Anxiety 10-40. |
| Hopkins Symptom Checklist-10 (HSCL-10) | Halvorsen et al. [32] | Not reported | 10 items. | Likert Scale. 1 = not at all to 4 = extremely | Not reported |
| Health Competence Beliefs Inventory (HCBI) | Brier et al. [30]  DeRosa et al. [31] | Not reported | 21; Health perceptions (6 items); Satisfaction with healthcare (5 items); Cognitive competence (5 items); Autonomy (5 items) | Checklist | Not reported |
| Perceived health competence scale | DeRosa et al. [31] | Not reported | Not reported | Not reported | Not reported |
| Metacognitions Questionnaire – 30 (MCQ-30) | Fisher et al. [33] | Not reported | 30 items (5 dimensions) | 4-point scale ranging from 1 (do not agree) to 4 (agree very much) | Subscale scores range from 6-24. Total scores range from 30-120 |
| Questionnaire on subjective wellbeing | Felder-Puig et al. [34] | Not reported | Not reported | 6-point scale | Not reported |
| State-Trait Anxiety Inventory – State subscale (STAI-S) | Santacroce et al. [26]  Felder-Puig et al. [34] | Not reported | 20 items | 4-point Likert scale | Not reported |
| Subjective Happiness Scale (SHS) | Bitsko et al. [42] | Present | 5 items | Not reported | Not reported |
| Center for epidemiologic studies depression (CES-D) | Bitsko et al. [42]  Langeveld et al. [52] | Not reported | 20 items | Not reported | Not reported |
| Hospital Anxiety and Depression Scale (HADS) | Fisher et al. [33]  Rae et al. 2020 [47] | 1 week | 14 items. 2 subscales (anxiety = 7 items, depression = 7 items) | 4-point Likert scale ranging from 0 = absence to 3 = extreme presence | 0-42 (0-21 per subscale) |
| Brief Symptom Inventory - 18 (BSI-18) | Brier et al. [30]  Zhou et al. [45] | Not reported | 18 items | Not reported | Produced a Global Severity Index (GSI) comprised of three subscales: depression, anxiety and somatic symptoms |
| Cancer Distress Scales for Adolescents and Young Adults (CDS-AYA) | Rae et al. 2020 [47]  Tsangaris et al. [48]  Rae et al. 2019 [49] | 1 week | 49 items across 5 scales | 4-point scale | 0-100 |
| Edmonton Symptom Assessment Scale – Revised (ESAS-r) | Rae et al. 2020 [47] | Present | 10 items | 11-point scale | 0-100 |
| Distress Thermometer | Rae et al. 2020 [47] | 1 week | 1 item | 0-10 | 0-10 |
| The Kessler Psychological Distress Scale (K10) | McCarthy et al. [53] | 4 weeks | 10 items | 5-point scale | 10-50 |
| Post Traumatic Stress Disorder Reaction Index | Santacroce et al. [26] | Not reported | 20 items | 5-point Likert scale | 12-59 |
| The Post Traumatic Growth Inventory (PTGI) | Santacroce et al. [26] | Not reported | 21 items | 6-point scale | Not reported |
| Growth Through Uncertainty Scale (GTUS) | Santacroce et al. [26] | Not reported | 39 items | 6-point scale | Not reported |
| The Impact of Events Scale – Revised (IES-R) | Fisher et al. [33] | 1 week | 22 items. 3 subscales (intrusions, avoidance and hyper-arousal) | 4-point Likert scale ranging from 0 (not at all) to 4 (extremely) | 0-88 |
| Post-Traumatic Stress Disorder Checklist – Civilian Version (PCL-C) | Taylor et al. [28]  Brier et al. [30]  DeRosa et al. [31] | 1 month | 17 items | 5-point Likert scale (1 = not at all, 5 = extremely) | 17–85 |
| Posttraumatic Stress Disorder Checklist (PCL-S) | McCarthy et al. [53] | 1 month | 17 items | 5-point Likert scale | 0-85 |
| The Post-Traumatic Diagnostic Scale (PDS) | Ganz et al. [44] | Varies from 1 week, 1 month, 3 months and 6 months | 39 items split into 3 sections | Checklist; scale 0-3; scale to indicate duration of symptoms | Not reported; 0-51; Not reported |
| PROMIS Self-Efficacy for Managing Symptoms Scale (PROMIS S-E) | Erickson et al. [27] | Not reported | 28 items | 5-point Likert scale ranging from “I am not confident at all” to “I am very confident” | Not reported |
| Chronic Disease Self-Efficacy Scale (Adapted) | Taylor et al. [28] | Not reported | 13 items | 5-point Likert scale (0-4 or not applicable) | Not reported |
| General Self-Efficacy Scale | DeRosa et al. [31] | Not reported | Not reported | Not reported | Not reported |
| Frankfurt Self-Concept Scale | Felder-Puig et al. [34] | Not reported | Not reported | 6-point scale | Not reported |
| Health Promoting Lifestyle Profile II (HPLP-II) | Santacroce et al. [26]  Finnegan et al. [43] | Not reported | 55 items | 4-point Likert scale | Not reported |
| Functional Assessment of Cancer Therapy – General Scale (FACT-G) | Skaczkowski et al. [25] | Not reported | Not reported | 5-point Likert scale ranging from “not at all” to “very much” | 0-108 |
| Paediatric Quality of Life Inventory (PedsQL) Young Adult version | Halvorsen et al. [32] | 1 month | 23 items grouped into 4 scales | 0-5 (0=never, 4=almost always) | 0-100 |
| Paediatric Quality of Life Inventory (PedsQL) 4.0 Generic Core Scales Adolescent Form | Ewing et al. [54] | 1 month | 23 items grouped into 4 scales | 0-4 (0=never, 4= almost always) | 0-100 |
| Paediatric Quality of Life Inventory (PedsQL) 3.0 Cancer Module Adolescent Form | Ewing et al. [54] | 1 month | 27 items grouped into 8 scales | 0-4 (0=never, 4= almost always) | 0-100 |
| Paediatric health-related quality of life (PedsQL) 4.0 | Bitsko et al. [42] | Not reported | Not reported | Not reported | Not reported |
| Questionnaire on Life Goals and Satisfaction with Life | Felder-Puig et al. [34] | Not reported | 3 items | 5-point scale | Not reported |
| Quality of Life – Cancer Survivors (QOL-CS) | Zebrack & Chesler [36] | Not reported | 41 items across 4 scales | 0-10 visual analogue scale | Average of 41 items |
| The Cancer Assessment for Young Adults – Testicular (CAYA-T) | Hoyt et al. [50] | Not reported | 90 items across 17 scales | 0-2 | Not reported |
| The Ferrans and Powers Quality of Life Index (QLI): Cancer III Versions | Finnegan et al. [43] | Not reported | Not reported | Not reported | 0-30 |
| Short Form-12 | Zhou et al. [45] | Not reported | 12 items | Not reported | Not reported |
| AYA Needs Assessment and Service Bridge | Haines et al. [35] | Current needs | 57 items across 9 domains | 3-point scale (agree, somewhat agree or not sure) | Not reported |
| Cancer Needs Questionnaire – Young People (CNQ-YP) | Clinton-McHarg et al. [37] | Time since diagnosis or 1 month | 139 items across 8 domains | 5-point scale | Not reported |
| Spinal Muscular Atrophy – Health Index (SMA-HI) | Mazzella et al. [58] | Not reported | 15 subscales each containing 5-19 questions | 7-point scale | 0-100 |
